# The integrative functional connectivity analysis between seafarer’s brain networks using functional magnetic resonance imaging data of different states

**DOI:** 10.3389/fnins.2022.1008652

**Published:** 2022-10-19

**Authors:** Yuhu Shi, Weiming Zeng

**Affiliations:** College of Information Engineering, Shanghai Maritime University, Shanghai, China

**Keywords:** fMRI, brain functional network, static functional connectivity, dynamic functional connectivity, affine propagation clustering

## Abstract

The particularity of seafarers’ occupation makes their brain functional activities vulnerable to the influence of working environments, which leads to abnormal functional connectivities (FCs) between brain networks. To further investigate the influences of maritime environments on the seafarers’ functional brain networks, the functional magnetic resonance imaging (fMRI) datasets of 33 seafarers before and after sailing were used to study FCs among the functional brain networks in this paper. On the basis of making full use of the intrinsic prior information from fMRI data, six resting-state brain functional networks of seafarers before and after sailing were obtained by using group independent component analysis with intrinsic reference, and then the differences between the static and dynamic FCs among these six brain networks of seafarers before and after sailing were, respectively, analyzed from both group and individual levels. Subsequently, the potential dynamic functional connectivity states of seafarers before and after sailing were extracted by using the affine propagation clustering algorithm and the probabilities of state transition between them were obtained simultaneously. The results show that the dynamic FCs among large-scale brain networks have significant difference seafarers before and after sailing both at the group level and individual level, while the static FCs between them varies only at the individual level. This suggests that the maritime environments can indeed affect the brain functional activity of seafarers in real time, and the degree of influence is different for different subjects, which is of a great significance to explore the neural changes of seafarer’s brain functional network.

## Introduction

Compared with the terrestrial environment, the working and living environment faced by seafarers at sea is quite different, so it is easy to influence the alterations of seafarers’ brain functional activities (Shi and Zeng, 2017, 2018).Therefore, it is necessary to explore the effects of this particular maritime environment on the brain functions of seafarers. Among them, functional magnetic resonance imaging (fMRI), as an emerging neuroimaging technology, is widely used in various researches on brain functional activities based on its superior performance in brain imaging (Poldrack et al., 2011). For example, Shi et al. first proposed a method for assessing psychological health of seafarers based on functional magnetic resonance imaging in 2015. By studying the functional connectivities (FCs) between the sub-regions of seafarers’ default mode network (DMN), they realized the binary classification of seafarers without labels through support vector machine. The statistical differences of the FCs of DMN between the two kinds of seafarers are further verified, which has attracted widespread attention from the industry (Shi et al., 2015b).

In fMRI studies, it is a mainstream method to investigate the law of cognitive development and neural mechanism of mental diseases through brain functional connectivities (FCs) analysis. Most studies on FCs mainly use various statistical methods to directly explore abnormal FCs and abnormal brain regions, while it is generally assumed that FCs are temporal-stationary in these studies, so that FCs are measured over the entire scan, and the brain network model is constructed as a static network using the entire period of the human brain time series data. This assumption provides a simple and convenient framework to examine large-scale brain networks and explore the correlation between functional and structural connectivity (Honey et al., 2009; Calhoun, 2018). For instance, Shirer et al. used the FCs between ninety functional regions of interest defined across 14 large-scale resting-state brain networks to decode the specific subject-driven cognitive states (Shirer et al., 2012). Moreover, Rosenberg et al. presented a broadly applicable neuromarker for sustained attention from whole-brain FCs (Rosenberg et al., 2016).

However, an increasing number of reports suggest that the FCs in the brain under both resting and task conditions are not stable, but show complex spatiotemporal dynamics due to the reasons that the brain undergoes dynamic integration, coordination, and responses to internal and external stimuli on multiple time scales (Calhoun et al., 2014; Calhoun and Adali, 2016; Preti et al., 2017). This phenomenon may mean that the time-varying dynamic FCs can offer a more complete description of brain activity than static FCs (Allen et al., 2014; Breakspear, 2017; Fu et al., 2017). Therefore, it is not enough to explain the time-varying and dynamic information interaction of the brain only considering static FCs when constructing the human brain network model. Instead, we should consider the dynamic nature of FCs and build a dynamic functional brain network, so as to better dig out the hidden information in the brain network, and then reveal the complex and changeable neural mechanism of the functional brain network (Damaraju et al., 2014; Yu et al., 2015; Tobia et al., 2017; de Vos et al., 2018; Shi et al., 2018).

Many studies have shown that dynamic FCs analysis can provide better evidence for pathologic inquiry and auxiliary diagnosis of clinical diseases as well as the neural basis of cognitive behaviors (Leonardi et al., 2013; Jia et al., 2014; Li et al., 2014; Hansen et al., 2015; Anees et al., 2017). For example, Robinson et al. presented a state-based dynamic community structure method to detect the time-dependent community structure in networks of brain regions. After applying the method, they found that the networks involved in pain, working memory, and emotion showed distinct profiles of time-varying connectivity (Robinson et al., 2015). Du et al. compared the dynamic FCs within DMN between healthy controls and schizophrenia patients using resting-state fMRI, and found that schizophrenia showed impaired interaction among DMN subsystems (Du et al., 2016). Furthermore, Faghiri et al. examined the relationship between age/maturity and the dynamics of brain FCs, realizing that dynamic FCs was an important factor to consider when examining brain development across childhood (Faghiri et al., 2018). Recently, dynamic FCs has been used to investigate the brain activity characteristics of specific occupational groups, such as taxi drivers and seafarers (Shen et al., 2016; Wang et al., 2017). In particular, Wang et al.’s study proved that sailors had one distinct atomic functional connectome compared to non-sailors, which may be likely linked to sailing experience. But little was researched on the influence of marine environment on seafarer’s brain functional activity from the perspective of intrinsic brain functional networks.

In this study, we are applying fMRI technology to explore the impact of maritime environment on the seafarer’s functional brain network before and after sailing, in which the functional brain networks detection is essential in fMRI data analysis. Better results can only be obtained in follow-up studies based on more prepared functional brain networks. Based on the above consideration, group independent component analysis (ICA) with intrinsic reference was employed for the functional brain networks detection by using prior information from fMRI data itself (Shi et al., 2015a), and then the differences between the static and dynamic FCs among these brain functional networks of seafarers before and after sailing were analyzed from both of group and individual levels, where the dynamic FCs among these functional brain networks were obtained using sliding time-window correlation. Subsequently, the dynamic patterns hidden in the dynamic FCs of seafarers before and after sailing were extracted by adopting affine propagation clustering (APC) algorithm and the transition probabilities between them were obtained simultaneously. The results showed the obvious changes between the brains functional connectivity networks of seafarers before and after sailing, which have a great value on exploring the neural rules of seafarer’s brain.

## Materials and methods

### Data acquisition

In this study, 33 seafarers from a shipping company of Shanghai participated and were collected the resting-state fMRI data before and after sailing, so the dataset included 66 fMRI data in total. The average time interval of them before and after sailing was about ten months. Before the data acquisition, all the participants were clearly informed about the purpose of this study and presented the written informed consent in accordance with the Declaration of Helsinki. In the process of data acquisition, all participants were instructed to keep the body motionless, eyes closed, relaxed (do not think anything systematically) and awake; their ears were stuffed up with the earplugs in order to reduce effect of the machine noise. The fMRI data of seafarers were acquired in the Shanghai Key Laboratory of Magnetic Resonance of the East China Normal University, and all procedures were approved by the Independent Ethics Committee of East China Normal University. The fMRI dataset was acquired on a Siemens 3.0 T scanner using a gradient echo planar imaging with 36 slices of whole-brain coverage and 160 volumes, a time of repetition of 2.0 s and a scan resolution of 64 × 64. The in-plane resolution was 3.75 mm × 3.75 mm, and the slice thickness was 4 mm. The data obtained through above scheme and parameters has also been used in (Shi, 2020a).

### Data preprocessing

In the experiment, all of the fMRI data were preprocessed by using the DPARSF software^1^, and the preprocessing steps included slice timing, motion correction, spatial normalization and spatial smoothing with the Gaussian kernel set to 4 mm. In particular, group ICA (GICA) was implemented using FastICA algorithm (Shi et al., 2017) in the GIFT software (v2.0e)^2^. Moreover, ICASSO (Himberg et al., 2004) with 20 runs of ICA was used to obtain reliable independent components (ICs), and minimum description length (Li et al., 2007) was used to estimate the number of ICs. Furthermore, the location and display of these networks were assessed by using the MRIcro software^3^. The whole above preprocessed process has also been used in (Shi et al., 2019).

### Brain functional components acquisition

In this study, the temporal and spatial functional components functional components at the group level were firstly decomposed by GICA with intrinsic reference (GICA-IR) method, and then those corresponding to each subject in the group were obtained by the way of dual-regression. Supposing that there are T time points and V voxels for all K subjects in the group after normalization, and then ICA was implemented on each subject, which can be defined as:


(1) $${\text{X}}_{\text{i}}={\text{M}}_{\text{i}}{\text{S}}_{\text{i}}\left(\text{i}=1,2,\cdots ,\text{K}\right)$$

where X_i_ is an T×V fMRI observed data, S_i_ = (s^11^, s_12_,⋯, s_1Ni_)′ is a N_i_×V matrix, and each row represents an independent component (IC) of subject i.M_i_ = (m_11_, m_12_,⋯, m_1Ni_) is a T×N_i_ mixing matrix. Next, we denoted s_in_i__ (i = 1,2,⋯,K) as the *n*_*i*_ st IC, which is the source of interest for the subject i. The correspondence of ICs between different subjects can be measured using spatial correlation, and then principal component analysis is used to extract the spatial reference signals corresponding to the components of interest from the K×V matrix R consisted of all s_in_i__ (i = 1,2,⋯,K):


(2) $$\text{R}={\left({\text{s}}_{1\,{\text{n}}_{1}},{\text{s}}_{2\,{\text{n}}_{2}},\cdots ,{\text{s}}_{{\text{Kn}}_{\text{K}}}\right)}^{{}^{\prime }}$$

Then, the eigenvalue λ_k_(k = 1,2,⋯,K) such that λ_1_≥λ_2_≥⋯≥λ_k_≥0, and the corresponding eigenvectors e_k_(k = 1,2,⋯,K) of covariance matrix C = E[RR′] can be calculated, where e_k_ is a column vector of sizeK×1. Finally, the first principal component $\text{r}={\text{e}}_{1}^{{}^{\prime }}\,R$ of size 1×V is selected as the intrinsic reference for group data analysis in GICA, which can be defined as:


(3) $$\left({\text{X}}_{1};{\text{X}}_{2};\cdots ,{\text{X}}_{\text{K}}\right)=\text{MS}$$

where M is a KT×V group mixing matrix, S = (*s*_1_,*s*_2_,⋯,*s*_*N*_)′ is a N×V matrix in which each row represents a GIC, and N denotes the number of GICs. The solving of (3) can be modeled as a constrained optimization problem as follows:

Maximize J(s_i_) = {E[G(s_i_)]−E[G(v)]}^2^


(4) $$\mathrm{Subject}\,\mathrm{to}\,\text{g}\,\left({\text{s}}_{\text{i}}\right)=\varepsilon \,\left({\text{s}}_{\text{i}},\text{r}\right)-\text{ξ}\leq 0\,a\,n\,d\,\text{h}\,\left({\text{s}}_{\text{i}}\right)=\text{E}\,\left[{\text{s}}_{\text{i}}^{2}\right]-1=0$$

where s_i_ is the output signal, J(s_i_) is the contrast function used to measure the independence of s_i_, G(•) is a non-quadratic function, v is a Gaussian random variable. r is the reference, ε(s_i_,r) is a distance criterion, ξ is a threshold parameter which needs to limit the distance such that the desired output signal should be the only one satisfying the inequality constraint. The equality constraint h(s_i_) is used to compel the output signal have a unit covariance (Shi et al., 2017).

### Functional connectivity analysis

In this section, both static FCs (SFCs) and dynamic FCs (DFCs) among the functional brain networks of seafarers before and after sailing are analyzed at the group level and individual level, respectively, where the brain functional networks are obtained from section “Brain functional components acquisition.” Among them, SFCs are computed by using Pearson correlation, whether between group functional brain networks or individual functional brain networks, and then the differences of SFCs between seafarers before and after sailing are calculated by statistical analysis. In particular, differences between group levels are measured by the overall FCs among the group functional brain networks, and differences between individual levels are measured by the FCs between each pair of functional brain networks. Meanwhile, the DFCs between functional brain networks are obtained by using the sliding time window correlation method for group level and individual level, respectively, and the difference of DFCs between each pair of functional brain networks of seafarers before and after sailing are statistically compared. The functional connectivity analysis can be implemented on brain connectivity toolbox^4^, and the detailed calculation process is shown as follows:

Firstly, the time courses of the six brain functional networks obtained from section “Brain functional components acquisition” are denoted as T_1_, T_2_,⋯,T_6_, and then the Pearson correlation coefficient (PCC) between pairwise brain functional networks are computed as SFCs. Secondly, the sliding window method with the window-width W of 20TRs and the step size of 1TR is used to compute the DFCs among the six brain functional networks, and the time course of the *n*th brain functional network in the *j*th time window is denoted as ${\text{DT}}_{\text{n}}^{\text{j}}\left(1\leq \text{n}\leq 6;1\leq \text{j}\leq \text{T}-\text{W}+1=141\right)$, so that 141 DFC matrices were obtained, and then these DFC matrices constituted the DFC matrix set DFCMS = {DFCM_1_, DFCM_2_,…, DFCM_j_,…, _DFCM141_}.

Among them, the PCC between the time course ${\text{DT}}_{\text{x}}^{\text{j}}$ and ${\text{DT}}_{\text{y}}^{\text{j}}$ of brain functional network x and y under the jth sliding window is calculated as follows:


(5) $${\text{corr}}_{\text{x},\text{y}}^{\text{j}}=\frac{\text{cov}\,\left({\text{DT}}_{\text{x}}^{\text{j}},{\text{DT}}_{\text{y}}^{\text{j}}\right)}{\sqrt{\text{var}\,\left({\text{DT}}_{\text{x}}^{\text{j}}\right)}\,\sqrt{\text{var}\,\left({\text{DT}}_{\text{y}}^{\text{j}}\right)}}$$

where $\text{cov}\,\left({\text{DT}}_{\text{x}}^{\text{j}},{\mathrm{DT}}_{\text{y}}^{\text{j}}\right)$ represents the covariance of ${\text{DT}}_{\text{x}}^{\text{j}}$ and ${\text{DT}}_{\text{y}}^{\text{j}}$ . $\text{var}\,\left({\text{DT}}_{\text{x}}^{\text{j}}\right)$ and $\text{var}\,\left({\text{DT}}_{\text{y}}^{\text{j}}\right)$ represent the variance of ${\text{DT}}_{\text{x}}^{\text{j}}$ and ${\text{DT}}_{\text{y}}^{\text{j}}$, in which 1 ≤ j ≤ 141,1 ≤ x ≤ 6,1 ≤ y ≤ 6.

Furthermore, the DFC matrix DFCM_j_ under the jth sliding window is composed of PCCs between the time courses of all brain functional networks, which are specifically expressed as:


(6) $${\text{DFCM}}_{\text{j}}=\left[\begin{array}{cccccc} {\text{corr}}_{1,1}^{\text{j}} & {\text{corr}}_{1,2}^{\text{j}} & \cdots & \cdots & \ldots & {\text{corr}}_{1,6}^{\text{j}}\\ {\text{corr}}_{2,1}^{\text{j}} & {\text{corr}}_{2,2}^{\text{j}} & \cdots & \cdots & \ldots & {\text{corr}}_{1,6}^{\text{j}}\\ \vdots & \vdots & \vdots & \vdots & \vdots & \vdots \\ {\text{corr}}_{\text{u},1}^{\text{j}} & {\text{corr}}_{\text{u},2}^{\text{j}} & \cdots & {\text{corr}}_{\text{u},\text{v}}^{\text{j}} & \ldots & {\text{corr}}_{\text{u},6}^{\text{j}}\\ \vdots & \vdots & \vdots & \vdots & \vdots & \vdots \\ {\text{corr}}_{6,1}^{\text{j}} & {\text{corr}}_{6,2}^{\text{j}} & \cdots & \cdots & \cdots & {\text{corr}}_{6,6}^{\text{j}} \end{array}\right]$$

where 1 ≤ j ≤ 141, 1 ≤ u ≤ 6, 1 ≤ v ≤ 6.

Next, the DFC matrix DFCM_j_ (1 ≤ j ≤ 141) in the DFC matrix set DFCMS is spanned into a column vector DFCV_j_ (1 ≤ j ≤ 141) with a size of 15×1 according to the triangular elements on the row of DFCM_j_, and then the 141 column vectors are cascaded according to the window time point from small to large, forming the DFC vector set DFCVS = [DFCV_1_, DFCV_2_, …,DFCV_j_,…, DFCV_196_] with a size of 141 × 15. Among them, DFCV_j_ refers to the DFC vector under the jth sliding window, which is specifically expressed as:


(7) $${\text{DFCV}}_{\text{j}}={\left[{\text{corr}}_{1,2}^{\text{j}},{\text{corr}}_{1,3}^{\text{j}},\cdots ,{\text{corr}}_{\text{u},\text{v}}^{\text{j}},\cdots ,{\text{corr}}_{5,6}^{\text{j}}\right]}^{{}^{\prime }}$$

### Affine propagation clustering

After obtaining the DFC vectors from section “Functional connectivity analysis,” APC is used to extract the implicit DFCs states from the DFCs of all seafarers before and after sailing as well as the DFCs of seafarers before and after sailing, respectively. In the APC algorithm, each data point is treated as an underlying clustering center, and the similarity s(*x*_*i*_,*x*_*k*_) (such as Euclidean distance) between the data point x_i_ and x_k_ is calculated, which is called preference parameter when i = k. The larger the value, the more likely the corresponding point is to be the candidate clustering center. In addition, two kinds of information transmission methods between data points are used: one is responsibility, denoted as α(i,k), which represents the fitness degree of data point x_k_ that can be used as the center of data point x_i_; the other is availability, denoted as α(i,k), which represents the fitness degree of data point x_i_ to choose data point x_k_ as its clustering center. It can be understood that the sum of responsibility of data point in the clustering center to other data points and the sum of availability of other data points to this data point are relatively large, so the possibility of this data point becoming the clustering center is relatively large. On the contrary, the sum of responsibility of data points at the edge of the cluster to other data points and the sum of availability of other data points to this data point are relatively small, so the possibility of this data point becoming the clustering center is relatively small. At the beginning of APC, the availability is first initialized to 0, that is α(i,k) = 0, and then the responsibility and availability are updated iteratively according to the following rules (Ren et al., 2014):


(8) $$\text{r}\,\left(i,k\right)\leftarrow \text{s}\,\left(\text{i},\text{k}\right)-\max_{{\text{k}}^{{}^{\prime }}\,s\,t,{\text{k}}^{{}^{\prime }}\neq \text{k}}\left\{\alpha \,\left(\text{i},{\text{k}}^{{}^{\prime }}\right)+\text{s}\,\left(\text{i},{\text{k}}^{{}^{\prime }}\right)\right\}$$

(9) $$\alpha \,\left(\text{i},\text{k}\right)\leftarrow m\,i\,n\,\left\{0,r\,\left(k\right)+\sum_{i^{{}^{\prime }}\,\,s\,\,t,\,i^{{}^{\prime }}\notin \left\{i,k\right\}}m\,a\,x\,\left[0,r\,\left(\,i^{{}^{\prime }},k\right)\right]\right\}$$

(10) $$\alpha \,\left(\text{k},\text{k}\right)\leftarrow \sum_{i^{{}^{\prime }}\,\,s\,\,t,\,i^{{}^{\prime }}\neq k}m\,a\,x\,\left\{0,r\,\left(\,i^{{}^{\prime }},k\right)\right\}$$

Finally, the best clustering center can be determined according to the sum of responsibility and availability of each data point, which is the DFC state, followed by the comparison of DFC states from seafarers before and after sailing.

### The computation of transition probability

In addition to the describing the connectivity differences that distinguish FC states, we can also examine the transitions between them. We can characterize transition behavior by considering FC as a Markov chain, a system that undergoes transitions between a discrete number of states (Allen et al., 2014). According to the results of APC, the DFC state corresponding to the DFC of each window can be determined. Thus, the DFCs corresponding to all windows can be described as a function that changes with time, and its function value is the corresponding DFC state. Then, the number of transitions from one DFC state to another state can be counted according to the memoryless property of Markov chain, and then divided by the window width –1 is the transition probability between these two states.

## Results and analysis

In this section, the results of SFCs and DFCs among the resting-state functional brain networks of seafarers before and after sailing are presented at the group and individual levels, respectively. Among them, the window width of 20TRs is used for the DFC analysis, so it contains 141 DFC networks. In particular, three different situations are considered when applying APC algorithm for the extraction of DFC states, namely obtained them from the DFCs of all seafarers before and after sailing as well as those of seafarers before and after, respectively. For presentation purposes, the seafarers before and after sailing are referred to as presailors and backsailors in the following, respectively.

**FIGURE 1:**
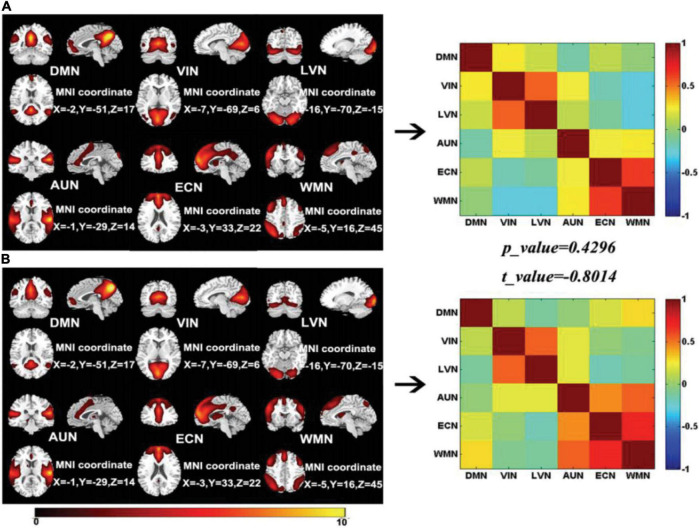
Six resting-state brain functional networks for seafarer before and after sailing at the group level, included DMN, VIN, ECN, AUN, LVN, and WMN which were shown in **(A,B)**, respectively, as well as the SFC maps of the two groups corresponding to the six brain functional networks and the T statistical test results between them.

Figure 1 shows six classical resting-sate brain functional networks obtained by GICA-IR for presailors and backsailors at the group level, included default mode network (DMN), visual network (VIN), lateral visual network (LVN), auditory network (AUN), executive control network (ECN), and working memory network (WMN), as shown in (A) and (B). The SFC maps among these six brain functional networks corresponding to the two groups of seafarers are also presented, and the statistical results of SFCs between the two groups are obtained through the paired *T*-test with a confidence level of 95%. It can be seen clearly from the figure that there is no significant difference between the SFCs of presailors and backsailors from the perspective of SFC analysis at the group level, which means that the SFCs among the six brain functional networks have no significant changes before and after sailing, i.e., marine environmental factors may not have a significant impact on them.

**FIGURE 2:**
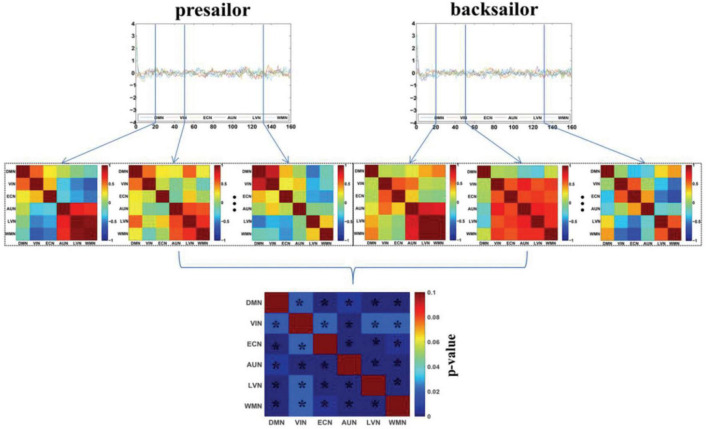
The DFC networks among the six resting-state brain networks of seafarers before and after sailing at the group level, as well as the statistical test results of differences between them. The asterisk indicates that there is a significant difference with a confidence level of *p* < 0.05.

**FIGURE 3:**
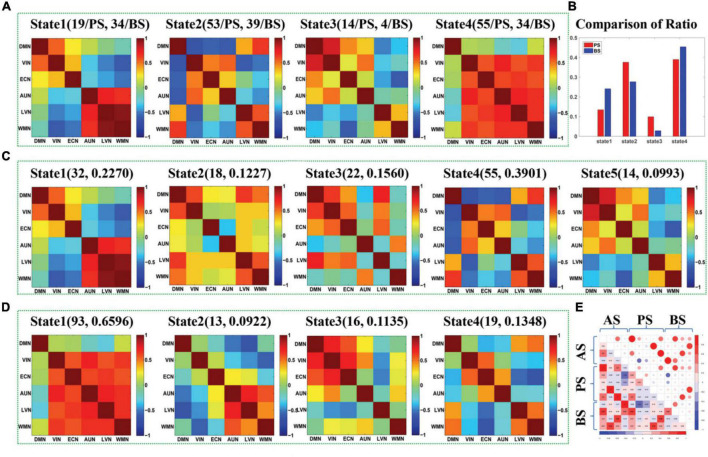
**(A)** The maps of four DFC states (State1-State4) extracted from the DFCs of presailors and backsailors at the group level, and the digits in parentheses on the top of each subfigure represent the number of DFCs contained in this state for presailors (PS) and backsailors (BS), respectively; **(B)** The histogram of proportion of the DFCs number corresponding to presailors and backsailors contained in each state of (A), respectively; **(C)** The maps of five states (State1-State5) extracted from the DFCs of presailors at the group level, and the digits in parentheses on the top of each subfigure represent the number and ratio of DFCs contained in this state; **(D)** The maps of four DFC states (State1-State4) extracted from the DFCs of backsailors at the group level, and the digits in parentheses on the top of each subfigure represents the number and ratio of DFCs contained in this state; **(E)** the correlation diagram between the three groups of DFC states in **(A,C,D)**.

Figure 2 shows the DFC networks among the six resting-state brain functional networks of seafarers before and after sailing at the group level, as well as the statistical test results of DFCs corresponding to each pair of brain networks between presailor and backsailor groups. According to the results of paired *T*-test results with a confidence level of 95% after false discovery rate (FDR) correction in the figure, there are significant differences in the DFCs between seafarers before and after sailing for each pair of the six resting-state brain functional networks involved in this study. This indicates that the FCs among the six resting-state brain networks have significant dynamic changes between the seafarers before and after sailing, which is inconsistent with the results of SFCs in Figure 1, so it is necessary to carry out further in-depth analysis, as shown in Figure 3.

Figure 3 presents the DFC states extracted by APC from the DFCs of all seafarers before and after sailing among the resting-state brain networks as well as those of presailors and backsailors extracted from the DFCs of seafarers before and after sailing at the group level, respectively. At the same time, we also present the number and ratio of DFCs contained in each DFC state, as shown in (A), (C) and (D), respectively. Figure 3B shows the proportion of DFCs contained in each state of (A) for seafarers before and after sailing respectively, as well as the statistical results after paired *T*-test with a confidence level of 95% between them. Figure 3E shows the correlations among the three groups of DFC states in (A), (C) and (D), respectively. As you can see from the figure that the DFC states obtained from the DFCs of three groups of different subjects corresponds to different FC situations between brain networks, reflecting the dynamic process of brain functional activities.

In addition, there is a high correlation between the DFC states extracted from the DFCs of all seafarers before and after sailing and the DFC states extracted from the DFCs of seafarers before and after sailing, respectively, as shown in Figure 3E. For example, state 1 in (A) and state 1 in (C), state 2 in (A) and state 4 in (C), state 3 in (A) and state 5 in (C), state 1 in (A) and state 2 in (D), state 2 in (A) and state 4 in (D), state 3 in (A) and state 3 in (D), and state 4 in (A) and state 1 in (D). It indicates that the dynamic characteristics of the brain network depicted by the total DFCs of two groups of seafarers and the DFCs of each group of seafarers are highly consistent. On the contrary, there is a weak correlation between the DFC states extracted from DFCs of seafarers before and after sailing separately, which the special living environment on the sea has a certain effect on the brain functional activities of seafarers, thus affecting the dynamic connectivity between brain functional networks. This is consistent with the results that the DFC states obtained from the DFCs of seafarers before and after sailing contains significant different number and proportion of DFCs for seafarers before and after sailing, as shown in Figure 3B.

**FIGURE 4:**
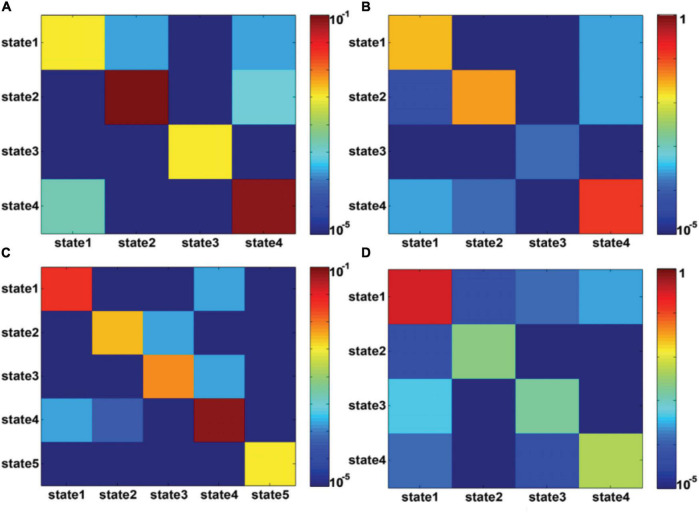
**(A,B)** represent the transition probability among DFC states for presailors and backsailors, where the DFC states extracted from the DFCs of all seafarers before and after sailing. **(C,D)** represent the transition probability among DFC states for presailors and backsailors, respectively, where the DFC states extracted from the DFCs of seafarers before and after sailing, respectively. Note that transition probability is color-mapped on a log-scale.

Figure 4 shows the state transition probabilities between the DFC states in Figures 3A,C,D, where (A) and (B) represent the state transition probability among the DFC states in Figure3A for seafarers before and after sailing, while (C) and (D) represent the transition probability between the DFC states in Figures 3C,D for seafarers before and after sailing, respectively. As you can see from the figure that the DFC states obtained from the DFCs of all seafarers before and after sailing at the group level present different state transition situations between seafarers before and after sailing, as shown in Figures 4A,B, respectively. For example, the probability of maintaining their own state is relatively balanced in presailor group, and the transition probabilities between different DFC states are relatively small; while the probability of different states remaining in their own state varies greatly backsailor group, in which the probability of state 4 is the largest, indicating that the brain functional connectivity network corresponding to this state plays an important role in seafarers’ navigation at sea. When DFC states are extracted from the DFCs of seafarers before and after sailing, the corresponding state transition situations show certain similarity. For example, state 1 has a high probability of maintaining in its own state. At the same time, it also shows some differences that the transfer probability between different DFC states is relatively large for seafarers before and after sailing, and this may be the result of rapid changes in the brain functional activity in the complex environment at sea.

**FIGURE 5:**
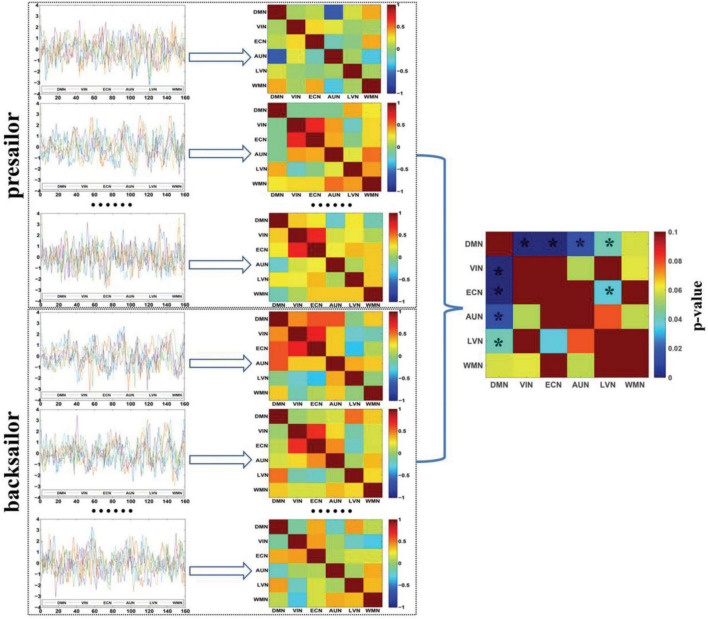
The SFCs among the six resting-state brain functional networks of seafarers before and after sailing at the individual level and the statistical analysis results of SFCs between seafarers before and after sailing for each pair of brain networks. The asterisk indicates that there is a significant difference with a confidence level of *p* < 0.05.

Figure 5 shows the SFCs of each seafarer before and after sailing among the brain functional networks at the individual level, and the statistical results of the SFCs difference corresponding to each pair of brain functional networks between seafarer before and after sailing. According to the results of paired *T*-test with a confidence level of 95% after FDR correction, the SFCs between DMN and the four brain networks of VIN, ECN, AUN and LVN, as well as the SFCs between ECN and LVN have significantly difference between seafarers before and after sailing. It indicates that the SFCs among several resting-state brain networks are significantly influenced by the maritime environment factors for some seafarers, which demonstrate the differences among individual subjects, so need more in-depth analysis.

**FIGURE 6:**
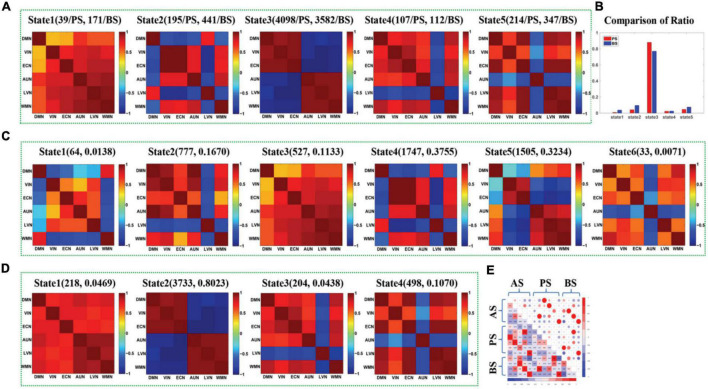
**(A)** The maps of five DFC states (State1-State5) extracted from the DFCs of presailors and backsailors at the individual level, and the digits in parentheses on the top of each subfigure represent the number of DFCs contained in this state for presailors (PS) and backsailors (BS), respectively; **(B)** The histogram of proportion of the DFCs number corresponding to presailors and back-sailors contained in each state of (A), respectively; **(C)** The maps of six states (State1-State6) extracted from the DFCs of presailors at the individual level, and the digits in parentheses on the top of each subfigure represent the number and ratio of DFCs contained in this state; **(D)** The maps of four DFC states (State1-State4) extracted from the DFCs of back-sailors at the individual level, and the digits in parentheses on the top of each subfigure represents the number and ratio of DFCs contained in this state; **(E)** the correlation diagram between the three groups of DFC states in **(A,C,D)**.

Figure 6 shows the DFC states extracted by APC from the DFCs of all seafarers before and after sailing among the sixresting-state brain networks as well as those of presailors and backsailors extracted from the DFCs of seafarers before and after sailing at the individual level, respectively. At the same time, we also present the number and ratio of DFCs obtained in each state, as shown in (A), (C) and (D), respectively. Figure 3B shows the proportion of DFCs obtained in each state of (A) for seafarers before and after sailing, respectively, as well as the statistical results after *T*-test with a confidence level of 95% between them. Figure 3E shows the correlation among three groups of DFC states in (A), (C) and (D), respectively.

It can be seen from the figure that the results are roughly similar to the results at the group level shown in Figure 3, except that the number of DFC states extracted from the DFCs of seafarers before and after sailing and the DFCs of seafarers before sailing are different due to individual differences and different DFCs quantity. In addition, the number of DFCs contained in state 3 is larger for both seafarers before and after sailing when using the DFCs of seafarers before and after sailing to derive DFC states at the individual level, which illustrates that this state reflects a kind of major brain functional connectivity network in seafarers’ brain functional activities. Moreover, there are significant differences between seafarers before and after sailing, indicating that the various influences of seafarers suffered at sea are mainly reflected in the brain functional connectivity network corresponding to this state, which plays an important role in seafarers’ career.

**FIGURE 7:**
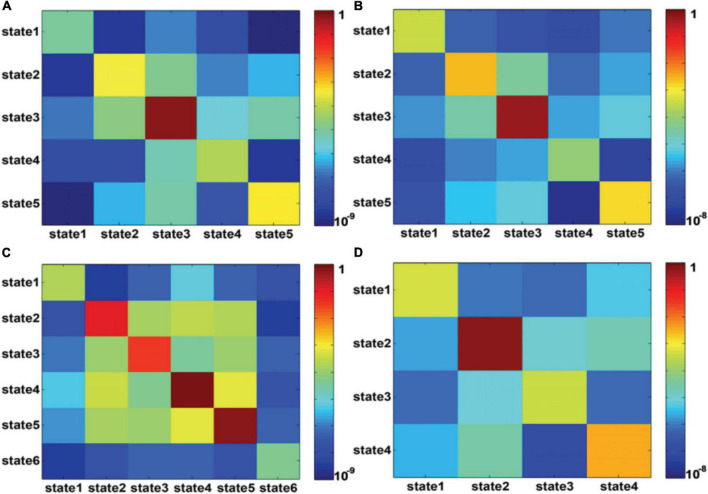
**(A,B)** represent the transition probability among DFC states for presailors and backsailors, where the DFC states obtained from the DFC matrices of all seafarers before and after sailing. **(C,D)** represent the transition probability among DFC states for presailors and backsailors, respectively, where the DFC states obtained from the DFC matrices of seafarers before and after sailing, respectively. Note that transition probability is color-mapped on a log-scale.

Figure 7 shows the state transition probabilities between the DFC states in Figures 6A,C,D, where (A) and (B) represent the state transition probability among the DFC states in Figure 6A for seafarers before and after sailing, while (C) and (D) represent the transition probability between the DFC states in Figures 6C,D for seafarers before and after sailing, respectively. As you can see from the figure that state 3 extracted from the DFCs of all seafarers before and after sailing at the individual level has a higher probability on their own status for seafarers before and after sailing, as shown in Figures 7A,B, respectively, which means that the corresponding brain functional connectivity network play an important role in the process of sailors at sea. Although the transition probabilities between different DFC states are relatively small, they are varying greatly between seafarers before and after sailing. This may be due to the reason that there are great differences when extracted the DFC states from DFCs of seafarers before and after sailing at the individual level, respectively. Firstly, the number of DFC states is different. Seafarers before sailing contain six states, while seafarers after sailing only contain four states. Among the six DFC states corresponding to seafarers before sailing, states 4 and 5 show a high probability of staying in their own state, while only state 2 show a high probability of staying in their own state for seafarers after sailing.

## Discussion

The previous studies using 88 seafarers before sailing showed that seafarers had significant differences in brain functional connectivity activity compared with non-seafarers, which may be due to the living and working environment faced by seafarers at sea (Shi and Zeng, 2017; Shi, 2020a,b; Shi et al., 2021b). However, since these seafarers had returned to land for a period of rest, whether these differences fully reflected the changes caused by the above environmental factors? In fact, by compared and analyzed the brain functional connectivity of seafarers before and after sailing at the voxel level, and found that there were significant differences between them (Poldrack et al., 2011; Shi et al., 2021a). This suggested that the differences in brain FCs of seafarers after returning to land for a period of rest cannot fully reflect the influences of maritime environments on the brain functional network of seafarers. In other words, some influences of maritime environmental factors on seafarers’ functional brain activities can be reversed, while others may have long-term effects, thereby reshaping the occupational brain plasticity of seafarers (Wang et al., 2017; Shi et al., 2019; Wu et al., 2020). However, this difference has not been considered at the level of large-scale brain network, which is exactly what this paper studies.

The 33 cases of brain imaging data involved in this study were obtained for the same seafarers before and after sailing. The difference was that they had worked at sea for an average of 10 months, so they had to face the special living and working environment at sea. According to previous studies (Poldrack et al., 2011; Shi et al., 2021a), these factors would affect their brain functional activities. Of course, this is a small amount of data, which is a limitation of this study. Therefore, it needed to obtain more such data to further expand and validate the findings of this paper. In addition, compared with DFCs, SFCs conducts the correlation analysis on the whole time series, so its sensitivity to time change is weaker than that of DFCs, which may be why there is no significant difference in SFCs among the brain networks between seafarers before and after sailing at the group level. However, the further study of this paper at the individual level found that these SFCs have significant differences between seafarers before and after sailing, which is consistent with the analysis results of DFCs.

The window length is a difficult parameter to select when using sliding window correlation method for dynamic functional connectivity analysis. If the selected window length is too short, it may increase the risk of introducing false fluctuation changes in DFCs analysis (Hutchison et al., 2013; Leonardi and Van De Ville, 2015; Zalesky and Breakspear, 2015), and the reliability of DFCs calculation appears to have too few samples simultaneously. Conversely, if the window length is too long, it is very likely that the DFC change modes of interest cannot be detected. According to previous studies, the implied cognitive status of DFCs can be correctly identified when the window length is 30s-60s (Shirer et al., 2012), so only the results on the window length of 40s were presented in this study. In this study, the FCs between six large-scale brain networks of seafarers before and after sailing are statistically analyzed, and it is found that there are significant differences between them, especially for DFCs. There are significant differences in DFCs between brain networks whether at the group level or at the individual level. This indicates that the FC patterns between brain functional networks have significantly variations during the voyage compared with those before sailing, and such variations will inevitably lead to changes in the potential stable patterns implied in DFCs, thus presenting different DFC states. This is why APC is used to mine different number of states from the DFCs of seafarers before and after sailing.

In order to explore the difference of stable DFC modes implied in the DFCs of seafarers before and after sailing, we extracted the potential DFC modes separately from the DFCs of all seafarers before and after sailing as well as those of themselves in the process of dynamic brain functional connectivity analysis, and then deeply analyze the DFC modes obtained from these three situations as well as the correlation between them. Moreover, it is different from the previous studies that usually adopt k-means clustering to obtain DFC modes, the APC algorithm was adopted to extract dynamic brain functional mode in this study. Since APC can determine the number of clustering adaptively, it overcomes the problem that the traditional k-means clustering method needs to select the number of categories in advance, thus reducing the adverse impact of subjective factors on extracting DFC modes.

The six resting state functional brain networks mentioned in this study (DMN, VIN, AUN, ECN, LVN, and WMN) are all related to the most basic cognitive functions of brain. Among them, DMN is associated with self-reflection, logical reasoning, emotional cognition, and interpersonal skills. While VIN and LVN play an important role in the recognition and judgment of basic features such as size, shape, color and position of objects, as well as the processing and feedback of complex objects. In comparison with non-seafarers, seafarers will face a variety of noises when navigating at sea and need to mobilize more auditory related areas, which will inevitably have some impact on their AUN. ECN is closely related to cognitive stimulation activities, such as making plans, making decisions, distinguishing right from wrong, responding to new situations, and overcoming habitual behaviors. It needs to be coordinated with the visual and auditory networks to play an important regulatory role in the networks associated with attention. WMN is primarily responsible for temporarily storing information that can be used for processing, which is important for guiding higher-level cognitive behavior such as reasoning and decision-making.

The results of this paper showed that there are significant differences between seafarers before and after sailing in the DFCs among the above six large-scale brain networks, both at the group level and at the individual level, and the number of potential DFC states in seafarers’ DFCs after sailing is less than that before sailing. For example, there are both four DFC states implied in the DFCs of seafarers after sailing at the group level and individual level, while there are five and six DFC states implied in the DFCs of seafarers before sailing at the group level and individual level, respectively. Moreover, the DFCs of seafarers after sailing are more concentrated in one of the DFC states, such as State3 at the individual level. This may be because seafarers are more focused during sea navigation and need to be ready to deal with emergencies at any time, and the patterns of processing information between brain networks are more stable. Of course, this effect may be different for each seafarer, which is consistent with the fact that the SFCs among brain networks show significant differences between seafarers before and after sailing at the individual level.

In this paper, we analyzed the functional connectivity between the large-scale brain networks of seafarers before and after sailing by fMRI data, and found that they have significantly changed before and after sailing, especially for DFCs, which were significantly different both at the group level and the individual level. Combined with previous studies (Shi and Zeng, 2017; Shi, 2020a,b; Shi et al., 2021b), the authors concluded that the influences of maritime environment on seafarers’ brain functional activities are time-dependent, some of which may cause permanent effects, while others can be recovered after a period of time. However, it contained many influencing factors due to the complex maritime environment, and the amount of data obtained at present is not large, so it is not possible to give definite conclusion for the moment. It can only serve as a preliminary exploration to lay a foundation for further in-depth research in the future, which is also the direction of our efforts.

## Conclusion

In this paper, on the basis of making full use of the intrinsic prior information of fMRI data, the SFCs and DFCs between the rsBFNs of seafarers before and after sailing are analyzed by GICA-IR method. The DFCs patterns hidden in the DFCs of seafarers before and after sailing are discovered by using the APC algorithm, and the probabilities of state transition between them are also obtained. The results show that the variations of seafarers’ functional brain connectivity networks before and after sailing may be time-dependent. It is of a great significance to explore the influence of marine environment on seafarers’ brain functional networks.

## Data availability statement

The raw data supporting the conclusions of this article will be made available by the authors, without undue reservation.

## Ethics statement

The studies involving human participants were reviewed and approved by the Independent Ethics Committee of East China Normal University. The patients/participants provided their written informed consent to participate in this study.

## Author contributions

YS and WZ: acquisition of fMRI data and analysis and interpretation. YS: design of the work and drafting the article. Both authors contributed to the article and approved the submitted version.

## Footnotes

1
http://rfmri.org/DPARSF

2
http://mialab.mrn.org/software/

3
http://www.mricro.com

4
https://sites.google.com/site/bctnet/
